# Long intergenic non‐coding RNA Linc00485 promotes lung cancer progression by modulating miR‐298/c‐Myc axis

**DOI:** 10.1111/jcmm.16036

**Published:** 2020-11-25

**Authors:** Zhenyang Zhang, Wenwei Lin, Yuhan Lin, Mingqiang Kang, Jiafu Zhu, Zhangwei Tong, Long Wu, Jianhai Sun, Jiangbo Lin

**Affiliations:** ^1^ Department of Thoracic Surgery Fujian Medical University Union Hospital Fuzhou Fujian China; ^2^ School of Stomatology Fujian Medical University Fuzhou Fujian China; ^3^ Department of Pathology Fujian Medical University Union Hospital Fuzhou Fujian China; ^4^ Department of Oncology Hubei No. 3 People's Hospital of Jianghan University Wuhan Hebei China

**Keywords:** c‐Myc, Linc00485, lung cancer, miR‐298

## Abstract

Long non‐coding RNAs (lncRNAs), which are non‐protein‐coding transcripts, are emerging as novel biomarkers for cancer diagnosis. Their dysregulation is increasingly recognized to contribute to the development and progression of human cancers, including lung cancer. Linc00485 is a newly discovered cancer‐related lncRNA; however, little is known about its role in lung cancer progression. In this study, we found that the expression of Linc00485 was significantly increased in human lung cancer tissue and associated with malignant phenotypes, including tumour‐node‐metastasis (TNM) stage, metastasis and relapse. Furthermore, the proliferative, migratory and invasive abilities of lung cancer cells in vitro were significantly enhanced by overexpression of Linc00485 but inhibited by its silencing. Mechanistically, Linc00485 regulated the expression of c‐Myc by directly binding to miR‐298; the effects of Linc00485 overexpression could be significantly reversed by a c‐Myc inhibitor or small interfering RNA. Xenotransplantation experiments showed that Linc00485 silencing significantly weakened the proliferation potential of A549 cells in vivo. Overall, these findings indicate that Linc00485 overexpression down‐regulates miR‐298, resulting in the up‐regulation of c‐Myc and thereby promoting the development of lung cancer.

## 1 INTRODUCTION

Lung cancer is a pulmonary malignancy with the highest morbidity and mortality among cancer‐related diseases worldwide.^1^ Although basic research and methods for clinical diagnosis and treatment have developed considerably over the past decades, most patients are diagnosed when already in the advanced stage of the disease, owing to the insidious symptoms of early‐stage lung cancer. Thus, the 5‐year survival rate of lung cancer patients is still very low.^2^, ^3^ Fully elucidating the mechanisms promoting the development and progression of lung cancer will enrich our understanding of tumour biology and help to find potential novel diagnostic and therapeutic targets for early‐stage lung cancer.

Non‐coding RNAs, which include microRNAs, long non‐coding RNAs (lncRNAs), P‐Element induced wimpy testis‐interacting RNAs, and circular RNAs (circRNAs), have traditionally been considered to be non‐functional transcriptional by‐products.^4^ However, recent studies have shown that non‐coding RNAs have important biological functions in cells.^5^, ^6^ MicroRNAs and other small RNAs modulate gene expression by binding to untranslated regions (UTRs) of mRNA.^4^, ^7^ LncRNAs^8^, ^9^ and circRNAs^10^ indirectly affect gene expression by functioning as sponges for microRNAs. Previous studies have suggested that lncRNAs have important roles in various types of cancer.^11^, ^12^, ^13^, ^14^ LincRNAs, a type of lncRNA, have been implicated in the initiation and progression of ovarian, pancreatic, and lung cancer.^14^, ^15^, ^16^, ^17^ Linc00152 was shown to promote the proliferation of colorectal cancer by regulating the expression of miR‐139‐5p, which is associated with metastasis and chemotherapy resistance.^18^ Linc00284 knockdown up‐regulated MEST expression by recruiting NF‐κB1, reducing angiogenesis in ovarian cancer and restraining cancer cell migration and invasion.^15^ We recently showed that Linc00485 was significantly up‐regulated in lung cancer tissues compared with adjacent normal tissues, and that it had a significant positive correlation with the expression of the c‐Myc oncogene.

The aim of the present study was to investigate the biological function of Linc00485 in lung cancer development, thus identifying a potential target for the diagnosis and treatment of lung cancer.

## 2 MATERIALS AND METHODS

### 2.1 Human lung cancer samples

Forty‐nine paired lung cancer tissues and adjacent normal tissues were collected from Fujian Medical University Union Hospital. None of the patients who provided samples had undergone preoperative chemoradiotherapy, and their lung cancer was not accompanied by other disease. Twenty‐nine patients were male and 20 were female, with an average age of 56.40 ± 7.10 years. There were 26 cases of lung adenocarcinoma and 23 cases of lung squamous cell carcinoma. Regarding TNM staging, 14 cases were stage I, 18 cases were stage II, and 17 cases were stage III. Further details are given in Table 1. The pathology of tumour tissues was strictly diagnosed by two independent expert pathologists. Tissues were immediately stored in liquid nitrogen at −196°C after surgery. All patients provided informed consent. The study was approved by the Ethics Committee of Fujian Medical University Union Hospital.

**TABLE 1:** Clinicopathological characteristics of lung cancer patients (n = 49)

Characteristics	Number of cases (%)
Age (y)	
≤60	18 (36.7)
>60	31 (63.3)
Gender	
Male	29 (59.2)
Female	20 (40.8)
Smoking	
Yes	37 (75.5)
No	12 (24.5)
Tumour invasion depth	
T1‐2	25 (51.0)
T3‐4	24 (49.0)
Tumour‐node‐metastasis staging	
Ⅰ+Ⅱ	32 (65.3)
Ⅲ+IV	17 (34.7)
Lymph node metastasis	
N0	11 (22.5)
N1 + N2	38 (77.5)
Distant metastasis	
M0	33 (67.4)
M1 + M2	16 (32.6)

### 2.2 Cell culture

Non‐small‐cell lung cancer (NSCLC) cell lines (A549 and H460 cells), human lung adenocarcinoma H1975 cells, lung epithelial BEAS‐2B cells, and 293T cells were purchased from the Cell Bank of the Chinese Academy of Sciences and maintained in our laboratory. A549 cells, H460 cells, and H1975 cells were cultured in RPMI1640 medium (HyClone) supplemented with 10% foetal bovine serum (Gibco) and 1% penicillin‐streptomycin (Invitrogen). BEAS‐2B cells and 293T cells were cultured using DMEM (HyClone) containing 10% foetal bovine serum and 1% penicillin‐streptomycin. All cells were cultured in saturated humidity and 5% CO_2_ at 37°C. The culture medium was changed every 24 hours.

### 2.3 Overexpression or knockdown of LincRNA 00485 in colorectal cancer cells

Cell transfection was performed using Lipofectamine 2000 (Invitrogen) according to the manufacturer's instructions, with si‐LincRNA 00485, si‐c‐Myc, mimics and antagomirs of miR‐298, pcDNA3.1‐LincRNA 00485 (overexpression of Linc00485; OE‐00485), pcDNA3.1‐c‐Myc (overexpression of c‐Myc; OE‐c‐Myc), and matched controls (all purchased from Guangzhou RiboBio Co., Ltd.). The concentration of RNA interference oligonucleotides for transfection was 2 µmol/L. After culturing for 48 hours, transfection efficiency was detected by quantitative polymerase chain reaction (qPCR) and/or western blotting. For animal experiments, lentivirus‐mediated short hairpin RNA was employed to knock down Linc00485 expression in the A549 cell line. Lung cancer cells were infected with the lentivirus (sh‐LincRNA 00485) (Guangzhou RiboBio Co., Ltd.) and then treated with puromycin (2 μg/mL) for 72 hours. The A549 cell line with stable silencing of Linc00485 was subcutaneously injected into nude mice. The transfection efficiencies are shown in Figures [Link], [Link], [Link].

### 2.4 Dual‐luciferase reporter assay

Wild‐type (WT) Linc00485 (WT‐00485), mutant Linc00485 (MUT‐00485), WT c‐Myc 3′‐UTR (WT‐c‐Myc), and mutant c‐Myc 3′‐UTR (MUT‐c‐Myc) plasmid vectors were constructed by Sangon Biotech (Shanghai) Co., Ltd. The relative luciferase activities of the co‐transfected miR‐298 mimic/inhibitor and WT/MUT 3′‐UTR expression vectors were evaluated by dual‐luciferase reporter assay 48 hours post‐transfection. Experiments were repeated independently at least three times.

### 2.5 Cell counting kit‐8 assay

Cells in logarithmic growth phase were seeded into 96‐well plates at a density of 10^5^ cells/well. Six replicate wells were used for each group. After 12 hours, 10% Cell counting kit‐8 (CCK‐8) (Beyotime Biotechnology Co.) was added to the cells at various time points following incubation for 2 hours. The absorbance at 450 nm was measured using a microplate reader (Bio‐Rad Laboratories). Culture medium supplemented with CCK‐8 reagent was used as a negative control. The experiment was repeated independently at least three times.

### 2.6 Flow cytometry for Ki‐67 staining

Cells in logarithmic growth phase were digested and resuspended at a concentration of 1 × 10^6^ cells/mL in phosphate‐buffered saline (PBS). After washing twice with PBS, the cells were fixed with methanol for 30 minutes and then treated with 1% Triton100 (Sangon Biotech) for 30 minutes at room temperature and blocked with goat serum (Sangon Biotech) for 1 hours. Subsequently, the cells were incubated with primary antibody Ki‐67 rabbit mAb (#9129; 1:400 dilution; Cell Signaling Technology Inc.) at 4°C for 10 hours, followed by incubation with fluorescein isothiocyanate‐conjugated fluorescent secondary antibody anti‐rabbit IgG (H + L), F(ab')2 Fragment (Alexa Fluor^®^ 488 Conjugate) (#4412, at 1:500 dilution; Cell Signaling Technology) for 2 hours at room temperature. Flow cytometry was used to detect the proportion of Ki‐67‐positive cells in each group. Experiments were repeated independently at least three times.

### 2.7 Scratch wound assay

As described previously,^19^ cells were plated into six‐well dishes with a density of 2 × 10^6^ cells per well. When the cells reached 90% confluence, a gap in the confluent monolayer was created using a 200 μL sterile pipette tip. The detached cells were removed using PBS. The wound healing process was recorded and photographed under a microscope every 12 hours. The experiment was repeated independently at least three times.

### 2.8 Transwell migration and invasion assays

Cells were collected, resuspended using serum‐free medium, and diluted to a final concentration of 1 × 10^5^ cells/mL. Then, 200 μL of the cell suspension was added into the upper compartments of 24‐well transwell dishes (8‐μm pore) (Corning). To detect the invasion of lung cancer cells, the upper compartment was pre‐coated with Matrigel (1 mg/mL) (BD Biosciences) for 1 hour at 37°C. The lower chamber contained 400 μL of complete medium. The cells were cultured for 12 hours (37°C, 5% CO_2_, saturated humidity). Cells that migrated to or invaded the lower compartment were fixed with 75% ethanol for 10 minutes, following staining by 0.1% crystal violet for 15 minutes. Non‐migrating or non‐invading cells were wiped gently with cotton swabs. Five fields of view from each well were photographed under a microscope. The protocols have been described in detail previously.^20^ The experiment was repeated independently at least three times.

### 2.9 Colony formation assay

Cells in logarithmic growth phase were collected and seeded into six‐well plates (1 × 10^3^ cells per well) and cultured for 10 consecutive days. Culture medium was refreshed every 48 hours. Subsequently, the cells were fixed with 75% ethanol for 30 minutes at room temperature and stained with 0.1% crystal violet for 20 minutes, followed by washing three times with PBS. Cells were counted for colonies containing at least 50 cells. Ten fields of view per group were observed and photographed under a microscope. The experiments were repeated independently at least three times.

### 2.10 qPCR analysis

Total RNA was isolated using TRIzol (Invitrogen) following standard procedures. Complementary DNA synthesis and qPCR were performed using PrimeScript^™^ RT Master Mix (Perfect Real Time) (Takara, #RR036A) and a One Step TB Green^®^ PrimeScript^™^ PLUS RT‐PCR Kit (Perfect Real Time) (#RR096A; Takara), respectively. The qPCR analysis was performed with an ABI 7500 FAST Real‐Time PCR system (Applied Biosystems). Housekeeping gene GAPDH was used to normalize gene expression. All primer sequences were synthesized by Sangon Biotech and are listed in Table 2. The experiments were repeated independently at least three times.

**TABLE 2:** Primers for real‐time PCR

Gene	Forward sequence	Reverse sequence
*Linc00485*	5′‐CTCCAAGCAGGGGCTACAAA‐3′	5′‐CCAGGAGCTCAGAAAGCCAA‐3′
*GAPDH*	5′‐CCCACTCCTCCACCTTTGAC‐3′	5′‐ATGAGGTCCACCACCCTGTT‐3′
*CDK4*	5′‐CTGGTGTTTGAGCATGTAGACC‐3′	5′‐GATCCTTGATCGTTTCGGCTG‐3′
*CDK6*	5′‐TCTTCATTCACACCGAGTAGTGC‐3′	5′‐TGAGGTTAGAGCCATCTGGAAA‐3′
*Bcl‐2*	5′‐CCAGCGTATATCGGAATGTGG‐3′	5′‐CCATGTGATACCTGCTGAGAAG‐3′
*P53*	5′‐GTTTCCGTCTGGGCTTCTTG‐3′	5′‐CACAACCTCCGTCATGTGCT‐3′
*Bid*	5′‐ATGGACCGTAGCATCCCTCC‐3′	5′‐GTAGGTGCGTAGGTTCTGGT‐3′
*Bax*	5′‐CCCGAGAGGTCTTTTTCCGAG‐3′	5′‐CCAGCCCATGATGGTTCTGAT‐3′
*N‐Cad*	5′‐TCAGGCGTCTGTAGAGGCTT‐3′	5′‐ATGCACATCCTTCGATAAGACTG‐3′
*MMP‐9*	5′‐AGACCTGGGCAGATTCCAAAC‐3′	5′‐CGGCAAGTCTTCCGAGTAGT‐3′
*MMP‐2*	5′‐CCCACTGCGGTTTTCTCGAAT‐3′	5′‐CAAAGGGGTATCCATCGCCAT‐3′
*E‐Cad*	5′‐CGAGAGCTACACGTTCACGG‐3′	5′‐GGGTGTCGAGGGAAAAATAGG‐3′
*CK‐19*	5′‐ACCAAGTTTGAGACGGAACAG‐3′	5′‐CCCTCAGCGTACTGATTTCCT‐3′
*miR‐298*	5′‐AGCAGAAGCAGGGAGGTT‐3′	5′‐ATACCTCGGACCCTGCACTG‐3′
*c‐Myc*	5′‐GTCAAGAGGCGAACACACAAC‐3′	5′‐TTGGACGGACAGGATGTATGC‐3′
*U6*	5′‐ATTGGAACGATACAGAGAAGATT‐3′	5′‐GGAACGCTTCACGAATTTG‐3′

### 2.11 RNA immunoprecipitation assay

The RNA immunoprecipitation (RIP) assay was performed using a Magnetic RIP kit (Merck KGaA) according to the manufacturer's instructions. Briefly, the cells were collected, washed twice with cold PBS, and resuspended in an equal volume of RIP lysis buffer. Then, they were placed on ice for 5 minutes, centrifuged at 12 000× *g* and 4℃ for 10 minutes. Next, 100 μL of supernatant was added to 900 μL of RIP Immunoprecipitation Buffer following incubation overnight at 4°C. Magnetic beads containing the immunoprecipitated RNA‐protein complex were treated with proteinase K. The total RNA in the precipitate was extracted according to the instructions and prepared for subsequent qPCR analysis.

### 2.12 Western blotting

Whole‐cell lysates were prepared using pre‐cooled RIPA lysis buffer (Beyotime Biotechnology Co.) containing 1% phenylmethanesulfonyl fluoride (Beyotime Biotechnology Co.). Protein concentrations were quantified using a bicinchoninic acid protein assay kit (KeyGen Biotech). Proteins (30 μg per lane) were separated by 10%‐12% sodium dodecyl sulfate‐polyacrylamide gel electrophoresis and then transferred to polyvinylidene fluoride membranes (Millipore). Next, the membranes were incubated with the appropriate antibodies prior to blocking with 5% bovine serum albumin. Immunoreactive bands were visualized using a chemiluminescence kit (Millipore). Relative gene expression was normalized to the expression of β‐actin. The following antibodies were used: anti‐c‐Myc (#9402, 1:1000 dilution; Cell Signaling Technology); anti‐β‐actin (#8457, 1:1000 dilution; Cell Signaling Technology); and goat anti‐rabbit IgG H&L (HRP) (ab6721,1:5000 dilution; Abcam). The experiments were repeated independently at least three times.

### 2.13 Animal experiments

Twelve 6‐week‐old female BALB/C nude mice (18‐20 g) were purchased from the Animal Center of Xiamen University. A suspension of cells (0.2 mL per site) in physiological saline at a concentration of 10^7^ cells/mL was inoculated subcutaneously into the axillae of mice. The short diameter (a) and the long diameter (b) of tumours of tumour‐bearing mice in each group were measured with callipers every 7 days, and the tumour volume was calculated according to the formula *V* = (*a* ^2^ × *b*)/2. On day 35 after transplantation, all tumour‐bearing mice were killed. Tumours were removed and weighed, and the average tumour weight of each group was calculated (n = 6 mice in each group). Animal experiments in this study were approved by the animal ethics committee of Fujian Medical University Union Hospital.

### 2.14 Statistical analysis

Data were analysed using SPSS 21.0 statistical software and expressed as mean ± SD. Differences between two groups were analysed by Student's *t* test. Comparisons among multiple groups with uniform variance were performed using one‐way ANOVA with post hoc Fisher's least significant difference (LSD) test. *P*‐values less than 0.05 were considered to indicate statistical significance. Each experiment was repeated independently at least three times.

## 3 RESULTS

### 3.1 Linc00485 is up‐regulated in lung cancer tissues and predicts poor outcomes in patients with lung cancer

**FIGURE 1:**
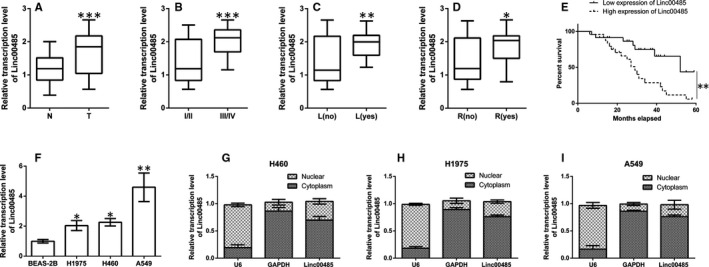
Linc00485 is up‐regulated in lung cancer tissues and associated with poor prognosis in patients with lung cancer. Expression levels of Linc00485 in (A) paracancerous tissues and tumour tissues of patients with lung cancer, (B) tumour tissues of patients with lung cancer at different TNM stages, (C) tumour tissues of patients with metastatic lung cancer, and (D) tumour tissues of patients with relapsed lung cancer. E, Association of Linc00485 with 5‐y survival of patients with lung cancer. F, Linc00485 levels in human bronchial epithelial cell line BEAS‐2B and different types of lung cancer cells. G‐I, Intracellular sub‐localization of Linc00485 in various lung cancer cell lines. Data are expressed as mean ± SD (n = 3). Differences between two groups were analysed by Student's *t* test. Comparisons among multiple groups with uniform variance were made using ANOVA with post hoc least significant difference test. L, lymphatic metastasis; N, adjacent normal tissues; R, relapse; T, tumour tissues. *P < 0.05, **P < 0.01, ***P < 0.001 compared with the control group

In this study, we found that the expression of Linc00485 in lung cancer tissues was significantly higher than that in adjacent normal tissues (Figure 1A). Linc00485 levels were associated with TNM stage in patients with lung cancer; the expression of Linc00485 was strikingly elevated in the tumour tissues of patients with stage III/IV lung cancer in comparison with those of patients with stage I/II lung cancer (Figure 1B). Moreover, Linc0048 levels were markedly increased in patients with lymphatic metastasis and in those with postoperative recurrent lung cancer, compared with those without metastasis or relapse (Figure 1C,D). Survival analysis suggested that patients with high expression of Linc00485 had significantly shorter survival times than those with low expression (Figure 1E). In vitro, the expression of Linc00485 was significantly up‐regulated in various cancer cells (H1975, H460, and A549) compared with human bronchial epithelial cell line BEAS‐2B (Figure 1F), and it was normally localized in the cytoplasm of tumour cells (Figure 1G‐I).

### 3.2 Linc00485 silencing inhibits and Linc00485 overexpression promotes proliferation of lung cancer cells

**FIGURE 2:**
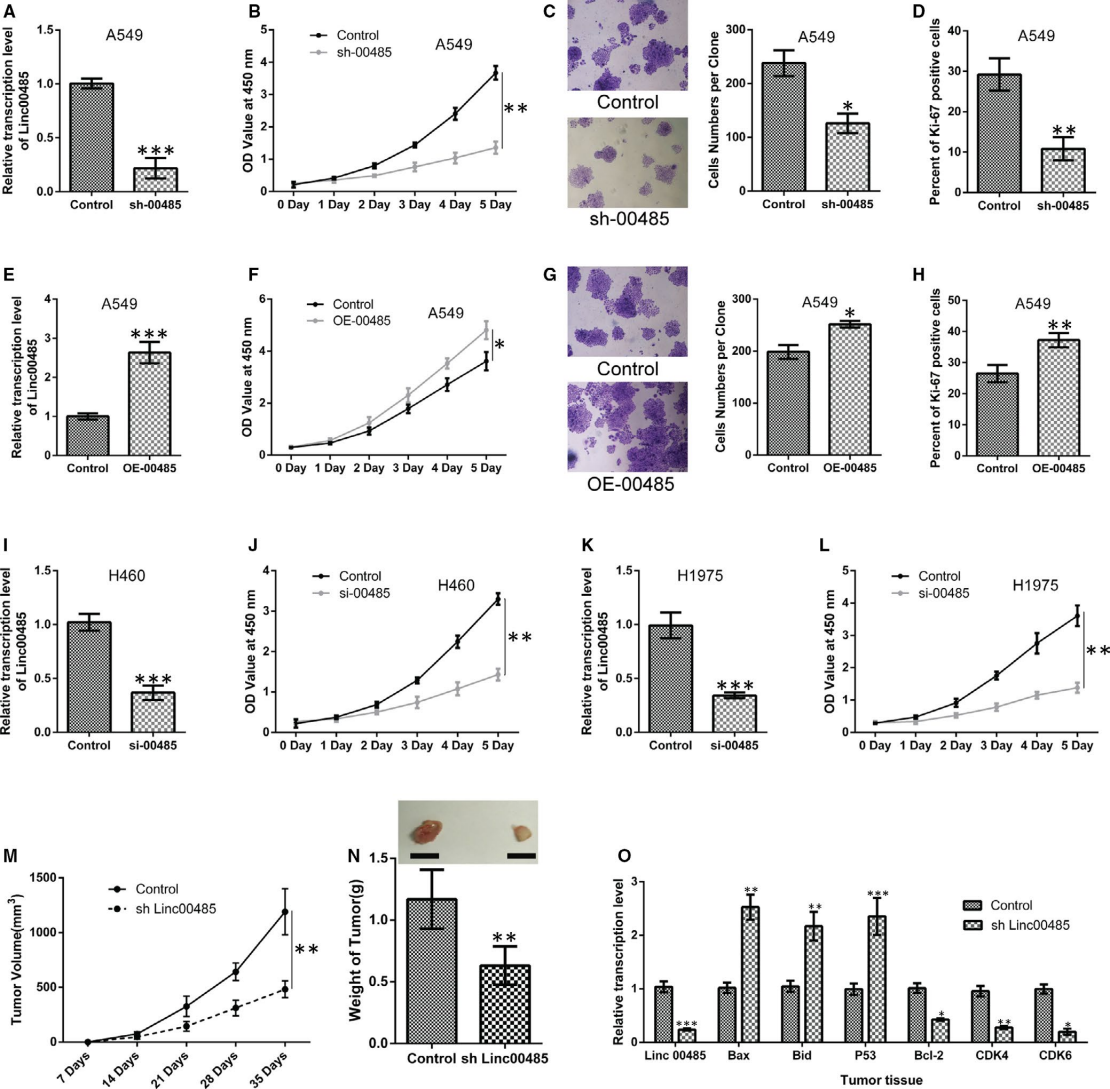
Linc00485 silencing significantly inhibited and Linc00485 overexpression promoted lung cancer cell proliferation. A, Efficiency of Linc00485 silencing as detected by quantitative polymerase chain reaction (qPCR) in A549 cells. B, Cell viability measured by Cell counting kit‐8 (CCK‐8) assay in A549 cells with Linc00485 silencing. C, Colony formation assay; the numbers of A549 cells in each colony were counted (n = 10 fields of view). D, Ki‐67‐positive rate of A549 cells with Linc00485 silencing, as detected by flow cytometry. E, Efficiency of Linc00485 overexpression, as detected by qPCR in A549 cells. F, Overexpression of Linc00485 promoted the viability of A549 cells. G, Cell number in each colony was significantly increased in A549 cells with Linc00485 overexpression. H, Linc00485 overexpression in A549 cells elevated the Ki‐67‐positive rate. I, Efficiency of Linc00485 silencing, as detected by qPCR in H460 cells. J, Linc00485 silencing significantly inhibited the viability of H460 cells. K, Efficiency of Linc00485 silencing, as detected by qPCR in H1975 cells. L, Linc00485 silencing markedly attenuated the viability of H1975 cells. M, Tumour volume was monitored weekly for 5 wk. N, Tumour weight was measured 35 d after implantation. O, Expression levels of cell proliferation‐related genes (Bax, Bid, P53, Bcl‐2, CDK4, and CDK6) in xenograft tumour tissues. Data are from three independent experiments and were analysed using Student's *t* test. Error bars represent SD. OE‐00485, overexpression of LincRNA 00485; sh‐00485, lentivirus‐mediated short hairpin RNA knockdown of LincRNA 00485; si‐00485, small interfering RNA for LincRNA 00485. *P < 0.05, **P < 0.01, ***P < 0.001 compared with the control group

To understand the role of Linc00485 in lung cancer progression, cells' proliferative ability was evaluated by CCK‐8 and colony formation assays and Ki‐67 staining. Our findings suggested that Linc00485 silencing significantly attenuated the viability of lung cancer cells (A549, H460, and H1975) (Figure 2A,B,I‐L), suppressed cell colony formation (Figure 2C), and decreased the Ki‐67‐positive rate in A549 cells (Figure 2D). By contrast, Linc00485 overexpression facilitated the proliferation of A549 cells (Figure 2E‐H). In a tumour xenograft animal model, the down‐regulation of Linc00485 (using short hairpin LincRNA 00485; sh‐00485) resulted in the inhibition of tumour growth of lung cancer compared with that in WT mice (n = 6 mice in each group) (Figure 2M,N). Notably, qPCR analysis revealed that Linc00485 silencing contributed to decreased expression of CDK4, CDK6, and Bcl‐2, and increased expression of P53, Bid and Bax in tumour tissues (Figure 2O).

### 3.3 Silencing of Linc00485 suppresses and overexpression of Linc00485 facilitates migration and invasion of lung cancer cells

**FIGURE 3:**
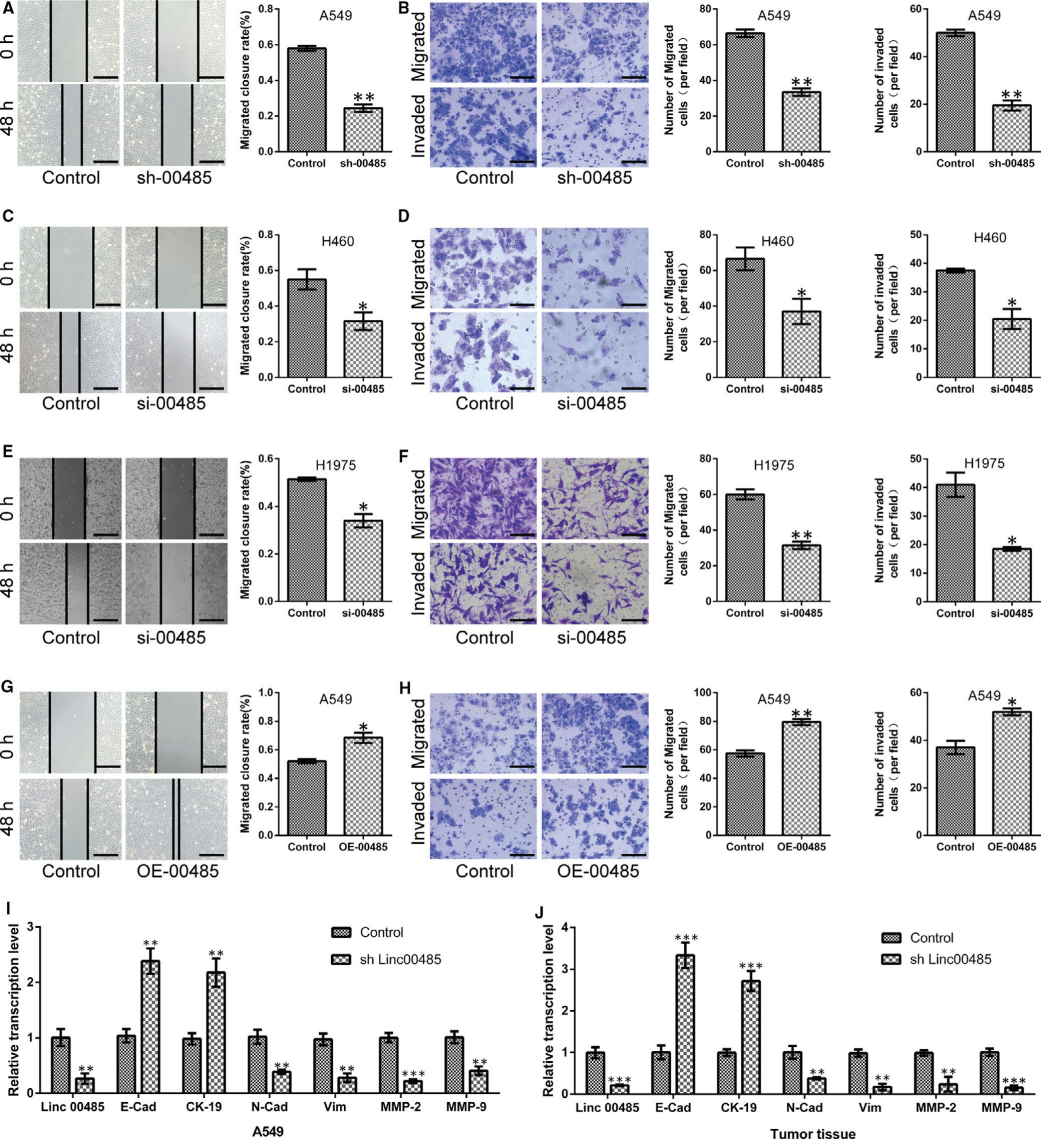
Silencing of Linc00485 suppresses and overexpression of Linc00485 facilitates migration and invasion of lung cancer cells. A‐F, Linc00485 silencing repressed the migration and invasion of (A, B) A549 cells, (C, D) H460 cells, and (E, F) H1975 cells. G and H, Linc00485 overexpression promoted the migratory and invasive abilities of A549 cells. I and J, Linc00485 levels and mRNA levels of E‐cadherin, CK‐19, N‐cadherin, vimentin, MMP‐2, and MMP‐9 in Linc00485‐silenced A549 cells and mouse tumour tissues (n = 6 mice in each group). Data are from three independent experiments and were analysed using Student's *t* test. Error bars represent SD. Scale bar = 50 μm. sh‐00485, lentivirus‐mediated short hairpin RNA knockdown of LincRNA 00485. *P < 0.05, **P < 0.01, ***P < 0.001 compared with the control group

Through in vitro scratch assays and transwell migration and invasion assays, we further confirmed that Linc00485 silencing significantly reduced the migrative and invasive capabilities of lung cancer cells (A549, H460, and H1975) (Figure 3A‐F), whereas the overexpression of Linc00485 significantly enhanced malignant phenotypes of A549 cells (Figure 3G,H). Epithelial‐mesenchymal transition (EMT) is a key biological process in metastasis, promoting tumour cell invasion and dissemination to distant organs.^21^ In the present study, the expression levels of EMT‐related genes were detected by qPCR. The silencing of Linc00485 significantly down‐regulated the expression of vimentin and N‐cadherin but up‐regulated that of E‐cadherin and cytokeratin 19 (CK‐19). Moreover, the expression levels of matrix metalloproteinase family members MMP‐9 and MMP‐2 were strikingly reduced in A549 cells with Linc00485 silencing (Figure 3I). Similarly, mRNA levels of vimentin, N‐cadherin, E‐cadherin, CK‐19, MMP‐9, and MMP‐2 in tumour tissues were consistent with the gene expression results in Linc00485‐silenced A549 cells (Figure 3J).

### 3.4 Linc00485 exerts its biological function by targeting miR‐298

**FIGURE 4:**
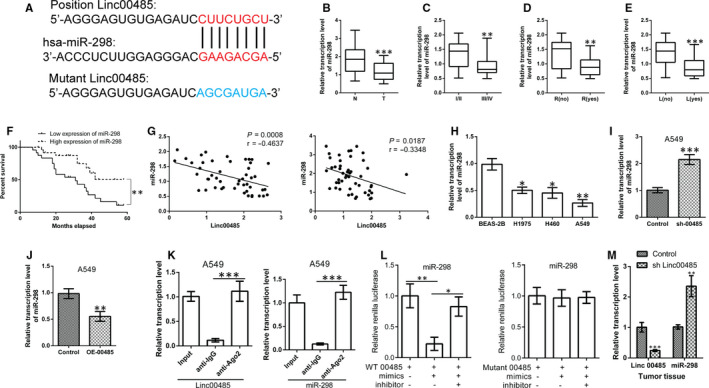
Linc00485 directly binds to miR‐298. A, Schematic of the binding sites between Linc00485 and miR‐298. B‐E, Expression levels of miR‐298 (B) in paracancerous tissues and tumour tissues of patients with lung cancer, (C) in patients with lung cancer at different TNM stages, (D) in patients with relapsed lung cancer, and (E) in patients with metastatic lung cancer. F, Lung cancer patients with low expression of miR‐298 had significantly better outcomes. G, Pearson correlation analysis showed that miR‐298 expression had a reverse correlation with Linc00485 expression in adjacent normal tissues (left) and tumour tissues (right) of patients with lung cancer, respectively. H, Expression levels of miR‐298 in lung epithelial cells and lung cancer cells. I and J, miR‐298 expression after (I) overexpression or (J) silencing of Linc00485 in A549 cells. K, RNA immunoprecipitation assay confirmed the interaction relationship between Linc00485 and miR‐298 in A549 cells. L, Dual‐luciferase reporter assay revealed that wild‐type (WT) Linc00485 (left) could bind to miR‐298, but mutant Linc00485 (right) could not. M, Expression of miR‐298 was significantly up‐regulated in xenograft tumour tissues with Linc00485 silencing (n = 6 mice in each group). Data are from three independent experiments and were analysed using Student's *t* test or ANOVA with post hoc least significant difference test. Correlation analysis was used to analyse the 5‐y survival of patients with lung cancer. Error bars represent SD. OE‐00485, overexpression of LincRNA 00485; sh‐00485, lentivirus‐mediated short hairpin RNA knockdown of LincRNA 00485. L, lymphatic metastasis; N, adjacent normal tissues; R, relapse; T, tumour tissues. *P < 0.05, **P < 0.01, ***P < 0.001 compared with the control group

By searching https://omictools.com/diana-lncbase-tool, we identified the sites of binding between miR‐298 and Linc00485 (Figure 4A). Expression of miR‐298 was found to be significantly decreased in human tumour specimens and lung cancer cells compared with normal tissues and normal lung epithelial cells, respectively (Figure 4B,H). Down‐regulation of miR‐298 expression was significantly associated with TNM stage, relapse, distant metastasis, and poor outcomes in patients with lung cancer (Figure 4C‐F); no correlations were observed between miR‐298 levels and patient gender, age, or pathological type (data not shown). Moreover, Pearson correlation analysis indicated that Linc00485 was negatively correlated with miR‐298 expression in adjacent normal tissues and tumour tissues of patients with lung cancer, respectively (Figure 4G). Silencing or overexpression of Linc00485 in A549 cells significantly increased or decreased expression levels of miR‐298, respectively (Figure 4I,J). Furthermore, the results of the RIP and dual‐luciferase reporter assays confirmed the direct interaction between Linc00485 and miR‐298 (Figure 4K,L). Notably, silencing Linc00485 up‐regulated the expression of miR‐298 in xenograft tumour tissues compared with the control group (n = 6 mice in each group) (Figure 4M).

### 3.5 Linc00485/miR‐298 axis regulates proliferation, migration, and invasion of lung cancer cells

**FIGURE 5:**
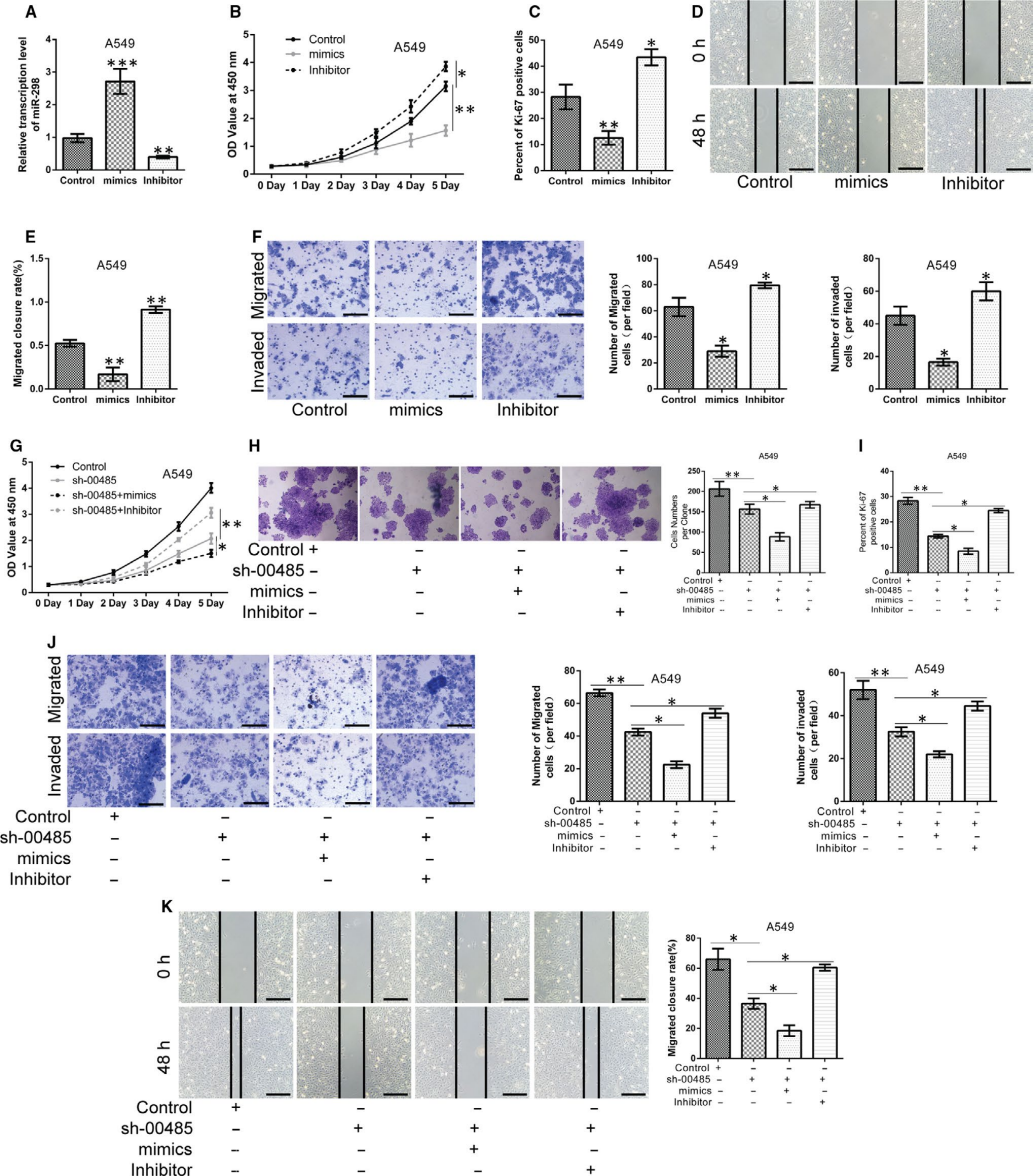
Linc00485/miR‐298 axis modulates proliferation, migration and invasion processes of lung cancer cells. A, Transfection efficiency of miR‐298 mimic or inhibitor in A549 cells. (B, C) Proliferation of A549 cells transfected with miR‐298 mimic or inhibitor measured by (B) Cell counting kit‐8 (CCK‐8) assay and (C) Ki‐67 staining. D‐F, The migratory and invasive capabilities of A549 cells transfected with miR‐298 mimic or inhibitor were evaluated by (D, E) an in vitro scratch assay, (F) transwell migration and invasion assays. G, CCK‐8 assay, (H) colony formation assays, and (I) Ki‐67 staining indicated that Linc00485 knockdown in combination with miR‐298 mimic transfection synergistically inhibited lung cancer cell viability, whereas miR‐298 inhibitor treatment could partially reverse the results of Linc00485 silencing. J and K, Cell motility and invasiveness were reduced in A549 cells after co‐transfection with sh‐00485 and miR‐298 mimic, whereas miR‐298 inhibitor reversed Linc00485‐knockdown‐induced changes compared with A549 cells treated with sh‐00485 alone. Data are from three independent experiments and were analysed using Student's *t* test or ANOVA with post hoc LSD test. Error bars represent SD. Scale bar = 50 μm. sh‐00485, lentivirus‐mediated short hairpin RNA knockdown of LincRNA 00485. *P < 0.05, **P < 0.01, ***P < 0.001 compared with the control group

To determine whether Linc00485 mediates the behaviours of lung cancer cells by sponging miR‐298, a mimic/inhibitor of miR‐298 or a negative control was transfected into A549 cells. We observed that down‐regulation of miR‐298 significantly enhanced the proliferative ability and elevated the Ki‐67‐positive rate of lung cancer A549 cells (Figure 5A‐C). Moreover, the migration and invasion abilities of A549 cells were significantly elevated by knockdown of miR‐298 (Figure 5D‐F). Conversely, up‐regulation of miR‐298 suppressed proliferation, migration, and invasion of lung cancer A549 cells compared with control cells. Furthermore, miR‐298 mimic transfection in combination with Linc00485 silencing had a synergistic inhibitory effect on the malignant behaviours of A549 cells in comparison with the cells treated with sh‐LincRNA 00 485 alone. However, the miR‐298 inhibitor partially reversed Linc00485 silencing‐induced changes in A549 cells (Figure 5G‐K). These results indicate that Linc00485 exerts its oncogenic activity in lung cancer by directly binding to miR‐298.

### 3.6 c‐Myc is the downstream target of the Linc00485/miR‐298 axis in lung cancer

**FIGURE 6:**
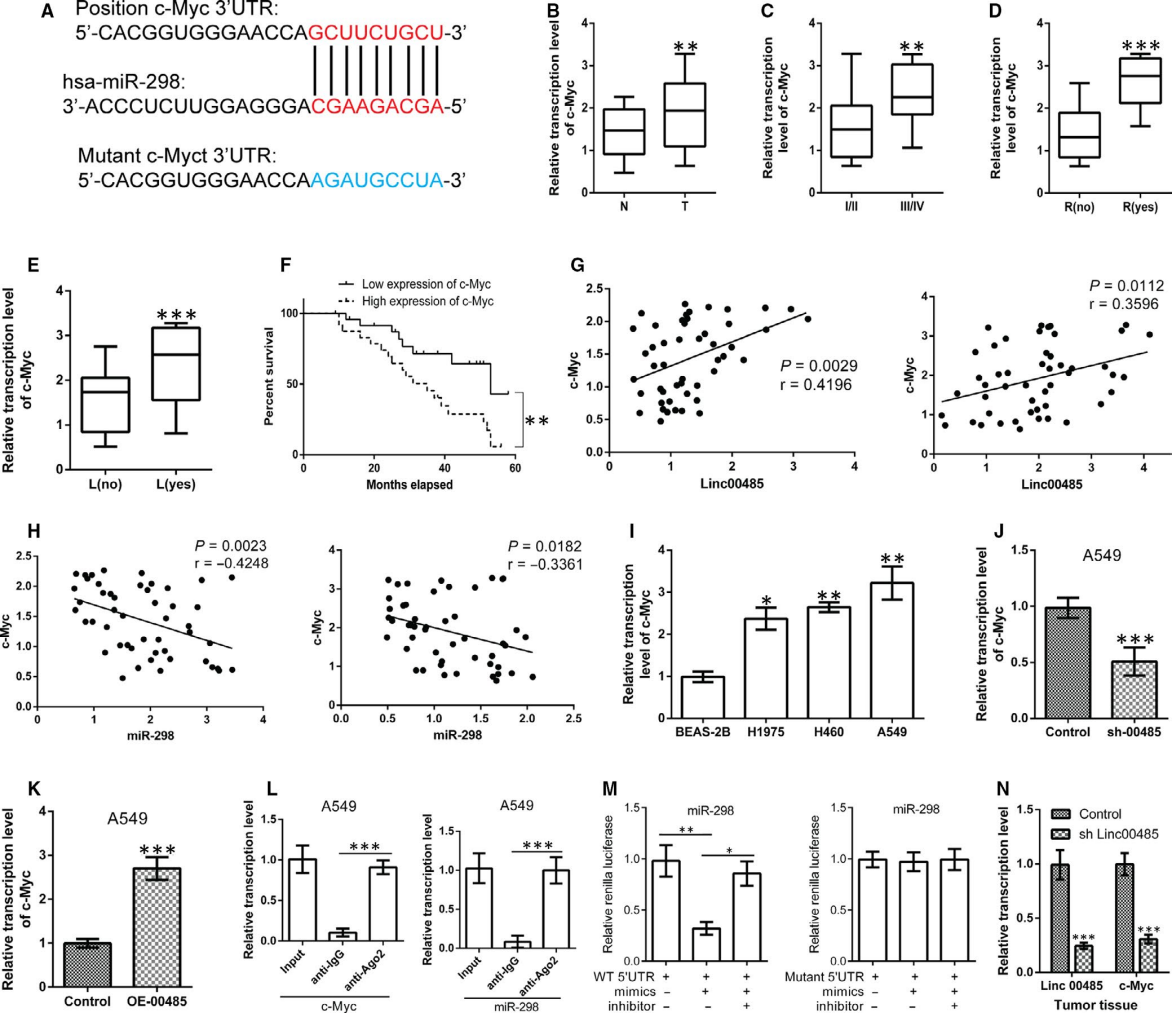
The c‐Myc gene is the downstream target of the Linc00485/miR‐298 axis. A, Schematic of the binding sites between miR‐298 and c‐Myc. B, Expression of c‐Myc in tumour tissues and paired normal tissues from patients with lung cancer, and its relationship with (C) TNM stage, (D) recurrence, (E) lymphatic metastasis and (F) 5‐y survival rate. G and H, Pearson correlation analysis indicated that (G) c‐Myc expression was positively correlated with the expression level of Linc00485 but (H) negatively correlated with miR‐298 levels in adjacent normal tissue (left) and tumour tissue (right). I, Expression levels of c‐Myc in lung epithelial cells and lung cancer cells. J, c‐Myc gene expression was dramatically down‐regulated in Linc00485‐silenced in A549 cells but (K) markedly up‐regulated in Linc00485‐overexpressing A549 cells compared with control cells. L, Interaction between c‐Myc mRNA (left) and miR‐298 (right) in A549 cells detected by RNA immunoprecipitation (RIP) assay. (M) Dual‐fluorescein reporter experiments confirmed that the wild‐type (WT) c‐Myc 3′‐untranslated region (UTR) (left) bound to miR‐298, but the mutant c‐Myc 3′‐UTR (right) could not. (N) Expression of c‐Myc in xenograft tumour tissues was significantly reduced after silencing of Linc00485 (n = 6 mice in each group). Data are from three independent experiments and were analysed using Student's *t* test or ANOVA with post hoc LSD test. Correlation analysis was used to analyse the 5‐y survival of patients with lung cancer. Error bars represent SD. L, lymphatic metastasis; N, adjacent normal tissues; R, relapse; T, tumour tissues. *P < 0.05, **P < 0.01, ***P < 0.001 compared with the control group

Further analysis using TargetScan^22^ indicated that miR‐298 may bind to the 3′‐UTR of c‐Myc mRNA (Figure 6A), regulating the expression level of c‐Myc. c‐Myc, a well‐known oncogene, controls a series of genes involved in tumour development, progression and metastasis. In our study, the c‐Myc gene was highly expressed in human tumour samples and lung epithelial cells compared with normal tissues and lung cancer cells, respectively (Figure 6B,I). Up‐regulation of c‐Myc was closely related to certain clinicopathological features, including tumour TNM stage, recurrence, distant metastasis, and survival time (Figure 6C‐F). Correlation analysis showed that the expression of c‐Myc was negatively correlated with miR‐298, and positively correlated with Linc00485 levels (Figure 6G,H). Transient silencing or overexpression of Linc00485 led to a significant down‐regulation or up‐regulation, respectively, of c‐Myc in A549 cells compared with control cells (Figure 6J,K). Moreover, miR‐298 overexpression strikingly down‐regulated the expression of c‐Myc in A549 cells (Figure S4). RIP and dual‐luciferase reporter assays confirmed the direct interaction between miR‐298 and c‐Myc mRNA (Figure 6L,M). We also tested the expression levels of c‐Myc in xenograft tissues. As expected, c‐Myc expression was markedly decreased in tumour tissues with Linc00485 knockdown compared with the control group (n = 6 mice per group) (Figure 6N).

### 3.7 Linc00485/miR‐298/c‐Myc axis is involved in the proliferation, migration, and invasion of lung cancer cells

**FIGURE 7:**
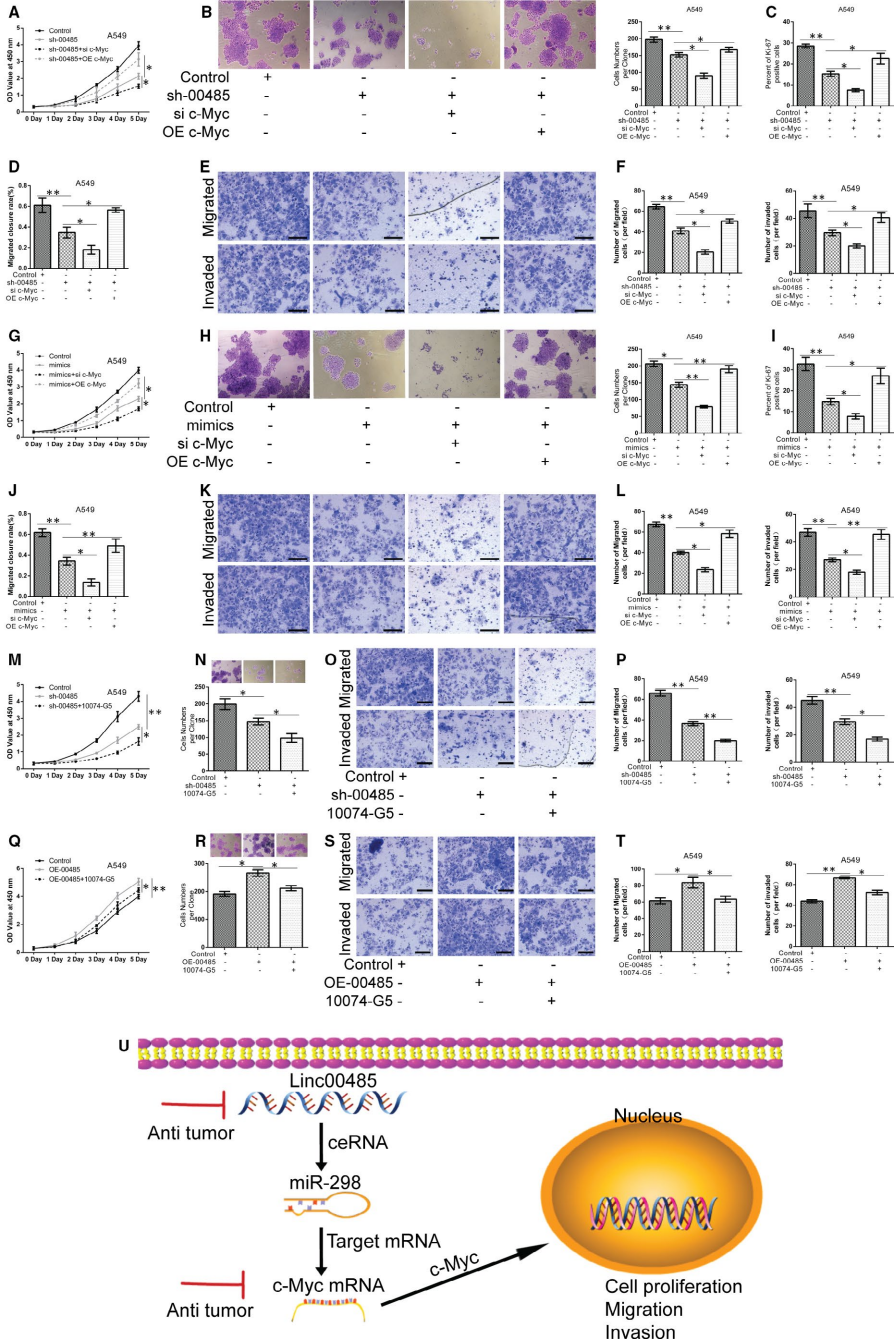
Linc00485/miR‐298/c‐Myc axis exerts pro‐tumour effects on proliferation, migration, and invasion of lung cancer cells. A‐C, Cell proliferation ability of A549 cells co‐transfected with sh‐00485 and si‐c‐Myc or OE‐c‐Myc, as tested by (A) CCK‐8, (B) colony formation assay, and (C) Ki‐67 staining. D‐F, Migratory and invasive ability of A549 cells co‐transfected with sh‐00485 and si‐c‐Myc or OE‐c‐Myc, measured using (D) an in vitro scratch assay, and (E, F) transwell migration and invasion assays. G‐I, Viability, (J‐L) migration, and invasion of A549 cells co‐transfected with miR‐298 mimic and si‐c‐Myc or OE‐c‐Myc. M‐P, Effects of Linc00485 silencing and c‐Myc inhibitor 10074‐G5 treatment (3 μmol/L, 24 h) or overexpression of c‐Myc on the proliferation, migration, and invasion of A549 cells. Q‐T, Effects of Linc00485 overexpression in combination with 10074‐G5 treatment (3 μmol/L, 24 h) on the proliferation, migration, and invasion of A549 cells. U, Schematic diagram illustrating the role of Linc00485 in lung cancer. Data are from three independent experiments and were analysed using ANOVA with post hoc LSD test. Error bars represent SD. Scale bar = 50 μm. OE‐00485, overexpression of LincRNA 00485; OE‐c‐Myc, overexpression of c‐Myc; sh‐00485, lentivirus‐mediated short hairpin RNA knockdown of LincRNA 00485; si‐c‐Myc, small interfering RNA for c‐Myc. *P < 0.05, **P < 0.01 compared with the control group

To elucidate the molecular mechanism by which the Linc00485/miR‐298/c‐Myc axis regulates lung cancer progression, the c‐Myc gene expression was silenced or over‐expressed, and cells were treated with the c‐Myc inhibitor 10074‐G5 (Sigma, 3 μmol/L) for 24 hours. Linc00485 silencing in combination with c‐Myc knockdown markedly attenuated the viability, migration, and invasion of lung cancer A549 cells, whereas overexpression of c‐Myc antagonized the effects of Linc00485 silencing (Figure 7A‐F). In addition, co‐treatment with miR‐298 mimic and si‐c‐Myc significantly inhibited the malignant behaviours of A549 cells compared with those transfected with miR‐298 mimic alone, whereas co‐treatment with miR‐298 mimic and pcDNA‐c‐Myc (overexpressing c‐Myc) produced the opposite results (Figure 7G‐L). Furthermore, silencing Linc00485 and treatment with c‐Myc inhibitor 10074‐G5 had stronger inhibitory effects on cell proliferation, migration, and invasion in comparison with silencing alone (Figure 7M‐P). Regardless of the overexpression of Linc00485, c‐Myc inhibitor 10074‐G5 treatment significantly suppressed the malignant behaviours of A549 cells (Figure 7Q‐T). Cumulatively, these data suggest that Linc00485 functions as an miR‐298 sponge to promote c‐Myc gene expression, resulting in proliferation, migration, and invasion of lung cancer cells (Figure 7U).

## 4 DISCUSSION

Accumulating evidence indicates that smoking, environmental pollution, diet and genetic susceptibility are responsible for the initiation and progression of lung cancer.^23^, ^24^ However, the pathogenesis of lung cancer has not yet been fully elucidated. LincRNA, a long‐chain non‐coding RNA, has been confirmed to be involved in cancer biology.^25^, ^26^, ^27^ In this study, we showed that the expression of Linc00485 was significantly up‐regulated in lung cancer tissues, suggesting that Linc00485 may be an oncogene.

Previous studies have found that Linc00485 plays an important part in the development of uterine leiomyoma.^28^ Moreover, in lung adenocarcinoma, Linc00485 up‐regulated the expression of cell cycle checkpoint kinase 1, thereby enhancing the resistance of lung cancer cells to chemotherapy.^29^ Here, our results confirmed that up‐regulation of Linc00485 in lung cancer tumour tissues and cells facilitated lung cancer cell proliferation, migration, and invasion. LincRNA exerts its biological function by adsorbing microRNAs.^18^, ^30^ Linc00485 could directly bind to miR‐298, leading to the up‐regulation of c‐Myc gene expression. In addition, patients with high expression of Linc00485 had significantly shorter survival times, suggesting that Linc00485 could be used as prognostic biomarker in patients diagnosed with lung cancer.

A large number of studies have demonstrated significantly decreased miR‐298 levels in different cancers, indicating that it acts as a tumour suppressor.^31^, ^32^, ^33^ Consistent with this, in this study, miR‐298 expression was found to be significantly down‐regulated in lung cancer tissues, and positively correlated with Linc00485. Using miR‐298 mimics not only inhibited the malignant characteristics of lung cancer cells but also partially reversed the phenotypes induced by Linc00485 overexpression.

c‐Myc is an oncogene that is significantly up‐regulated in various cancers, including lung cancer, and is associated with malignant phenotypes of cancer cells.^34^ Specific inhibitors of c‐Myc have been developed and have shown remarkable anti‐tumour activity in vitro and in vivo.^35^, ^36^ In our study, overexpression of Linc00485 up‐regulated c‐Myc expression. Furthermore, c‐Myc inhibitor treatment reversed the phenotypes resulting from Linc00485 overexpression. Taken together, this evidence suggests that Linc00485 modulates the expression of c‐Myc, thus participating in lung cancer progression. However, c‐Myc has previously been described as an upstream regulator of two well‐described lncRNAs, H19 and ANRIL (CDKN2B‐AS1).^37^ H19, a direct transcriptional target of MYC, was shown to be up‐regulated in NSCLC tumour tissues.^38^, ^39^, ^40^ CDKN2B‐AS1, which functions as an oncogene, was up‐regulated in NSCLC tumour tissues and cell lines.^41^ CDKN2B‐AS1 can be transactivated by c‐Myc, and its high expression promotes lung cell proliferation and migration.^42^ Therefore, we speculated that the overexpression of Linc00485 elevated the expression of c‐Myc by sponging miR‐298, thereby activating multiple downstream genes including H19 and ANRIL, and thus driving lung cancer development. The underlying mechanisms still require further clarification. Although our understanding of the role of Linc00485 in lung cancer progression is still in its infancy, there is no doubt that its further study will help us to find new targets for the treatment of patients with lung cancer.

Collectively, our findings reveal that Linc00485 regulates the expression of oncogene c‐Myc by adsorbing miR‐298. The Linc00485/miR‐298/c‐Myc axis is an important signalling pathway in the development of lung cancer, which may provide new insights into the pathogenesis of lung cancer and a potential target for diagnosis and treatment of lung cancer.

## CONFLICTS OF INTEREST

The authors confirm that there are no conflicts of interest.

## AUTHOR CONTRIBUTION

**Zhenyang Zhang:** Data curation (equal); Formal analysis (equal); Investigation (equal); Methodology (equal); Resources (equal); Software (equal); Writing‐original draft (equal); Writing‐review & editing (equal). **Wenwei Lin:** Data curation (equal); Investigation (equal); Methodology (equal); Resources (supporting); Software (supporting); Writing‐original draft (equal); Writing‐review & editing (equal). **Yuhan Lin:** Investigation (equal); Project administration (supporting); Software (supporting); Writing‐original draft (supporting); Writing‐review & editing (supporting). **Mingqiang Kang:** Investigation (supporting); Methodology (supporting); Project administration (supporting); Resources (equal); Software (supporting); Writing‐original draft (supporting); Writing‐review & editing (supporting). **Jiafu Zhu:** Investigation (supporting); Methodology (supporting); Project administration (supporting); Resources (supporting); Software (supporting); Writing‐original draft (supporting); Writing‐review & editing (supporting). **Zhangwei Tong:** Investigation (supporting); Methodology (supporting); Project administration (supporting); Resources (supporting); Software (supporting); Validation (supporting); Writing‐original draft (supporting); Writing‐review & editing (lead). **Long Wu:** Formal analysis (supporting); Investigation (supporting); Methodology (supporting); Resources (supporting); Software (supporting); Writing‐original draft (supporting); Writing‐review & editing (supporting). **Jianhai Sun:** Conceptualization (equal); Funding acquisition (equal); Resources (equal); Supervision (equal); Writing‐original draft (supporting); Writing‐review & editing (supporting). **Jiangbo Lin:** Conceptualization (lead); Funding acquisition (lead); Methodology (supporting); Supervision (lead); Writing‐original draft (supporting); Writing‐review & editing (supporting).

## ETHICAL APPROVAL

This study was approved by the ethics committee of Fujian Medical University Union Hospital, and written informed consents were obtained from patients enrolled in this study.

## Supporting information

Fig S1

Fig S2

Fig S3

Fig S4

Supplementary Material

## Data Availability

The data supporting the findings of this study are included within the article or are available upon request from the corresponding author.
