# Supplementary figures and images for: Long intergenic non‐coding RNA Linc00485 promotes lung cancer progression by modulating miR‐298/c‐Myc axis
Source: J Cell Mol Med. 2020 Nov 25;25(1):309–22. doi: 10.1111/jcmm.16036 (PMC7810966; doi:10.1111/jcmm.16036)

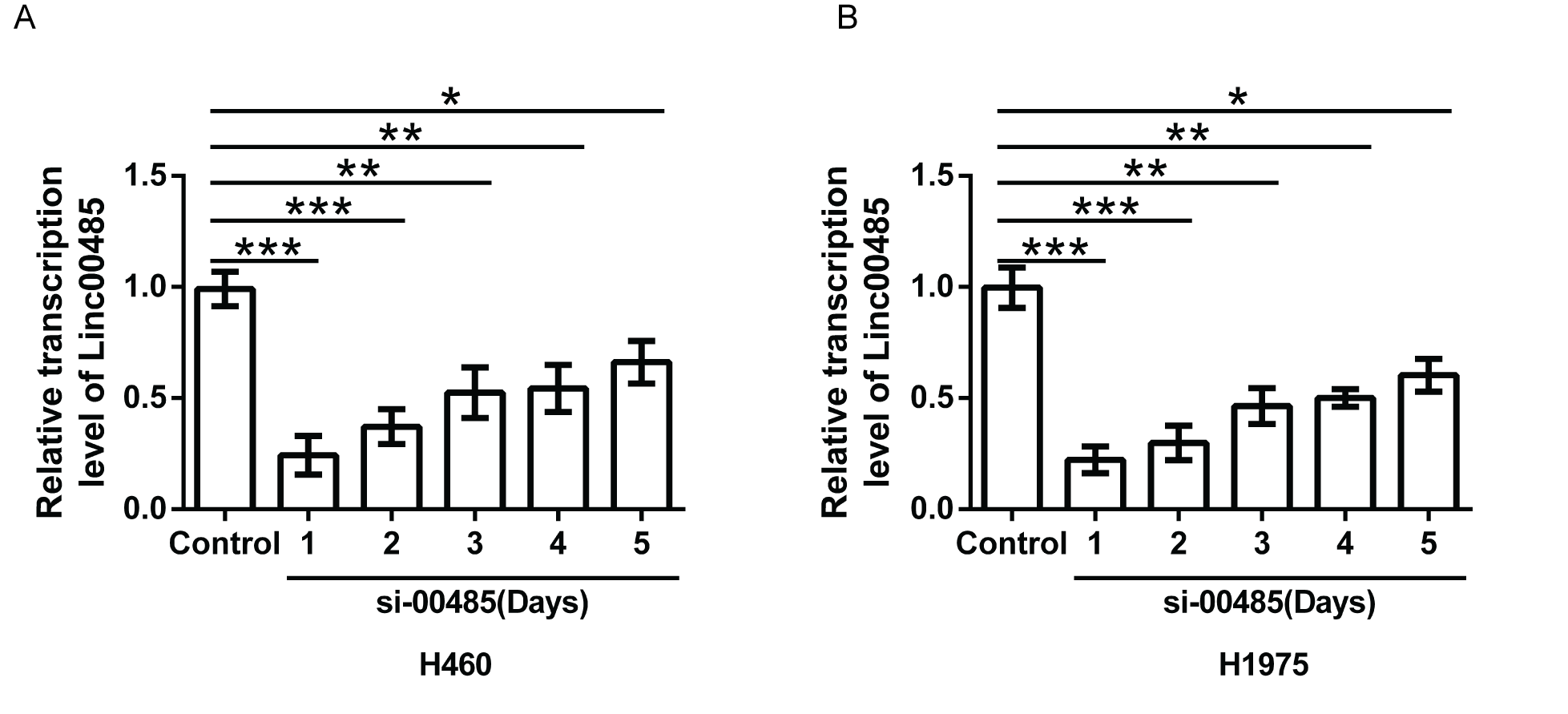

Supplement: Supplementary file 1 — Fig S1 [file JCMM-25-309-s001.tif]

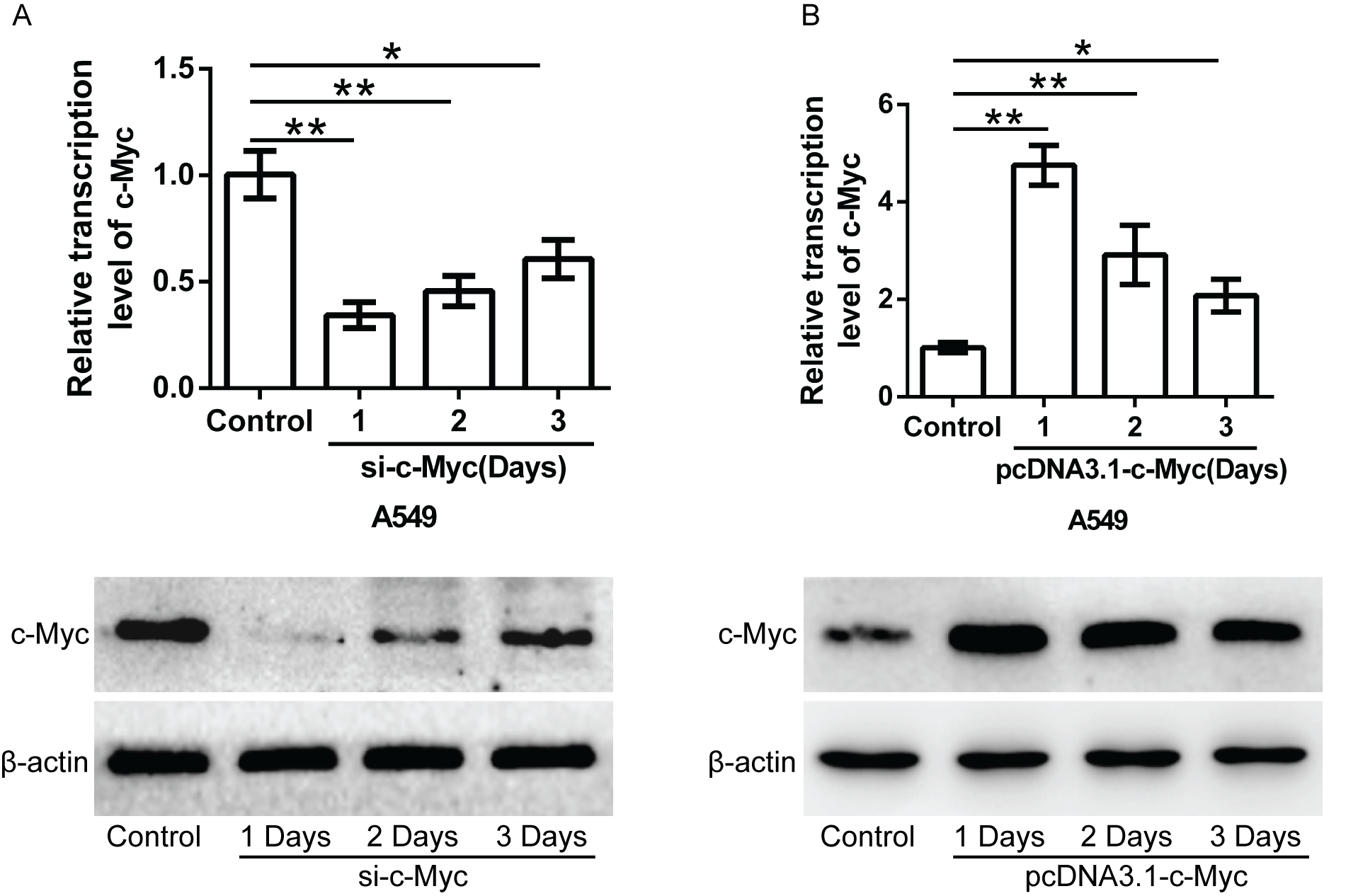

Supplement: Supplementary file 2 — Fig S2 [file JCMM-25-309-s002.tif]

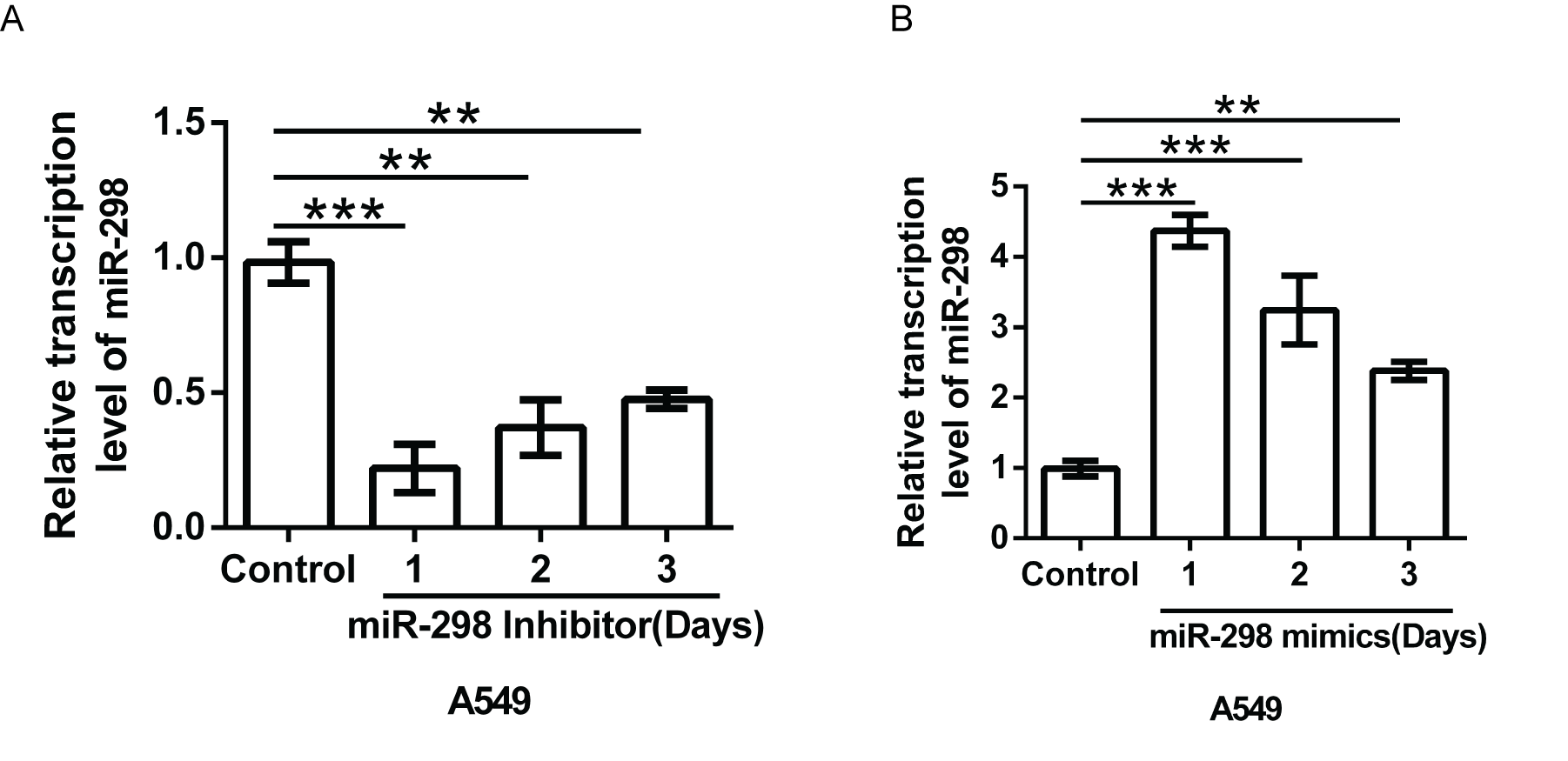

Supplement: Supplementary file 3 — Fig S3 [file JCMM-25-309-s003.tif]

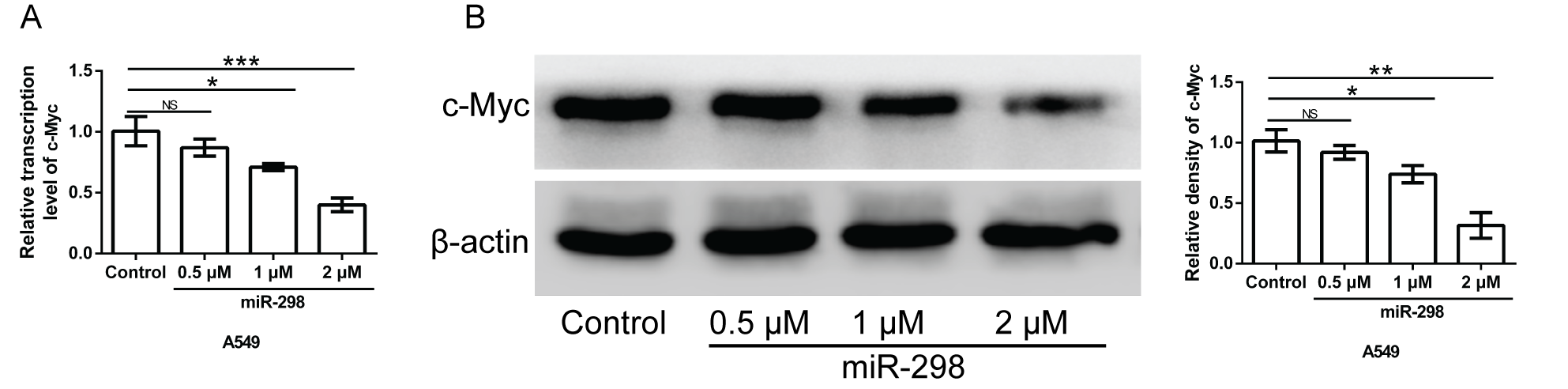

Supplement: Supplementary file 4 — Fig S4 [file JCMM-25-309-s004.tif]
