# Supplementary material for: Long intergenic non‐coding RNA Linc00485 promotes lung cancer progression by modulating miR‐298/c‐Myc axis
Source: J Cell Mol Med. 2020 Nov 25;25(1):309–22. doi: 10.1111/jcmm.16036 (PMC7810966; doi:10.1111/jcmm.16036)
Supplement: Supplementary file 5 — Supplementary Material [file JCMM-25-309-s005.docx]

Supplementary Figure 1: Transfection efficiency of si-Linc0048 and pcDNA-Linc00485 in various types of lung cancer cells. (A, B) Transient transfection efficiency of (A) si-00485 or (B) pcDNA3.1-00485 in A549 cells. (C, D) Transfection efficiency of si-00485 (C) in H460 cells (C) or (D) in H1975 cells. Data are presented as mean ± SD (n = 3). Data are from three independent experiments. *P*-values were calculated using ANOVA with post hoc LSD test. Error bars represent SD. * *P* < 0.05, ** *P* < 0.01, *** *P* < 0.001 compared with control group. si-00485, small interfering RNA for Linc 00485.

Supplementary Figure 2: The transfection efficiency of si-c-Myc and pcDNA-c-Myc in A549 cells. (A) The transient transfection efficiency of si-c-Myc in A549 cells (above) and the protein expression of c-Myc with time (below). (B) The transient transfection efficiency of pcDNA3.1c-Myc in A549 cells (above) and the protein expression of c-Myc with time (below). Data were from three independent experiments. *P*-values were calculated using ANOVA with post hoc LSD test. Error bars represented as SD * *P* < 0.05, ** *P* < 0.01, *** *P* < 0.001 as compared with the control group. si-c-Myc: small interfering RNA for c-Myc.

Supplementary Figure 3: Transfection efficiency of miR-298 inhibitor and mimic in A549 cells. (A) Transient transfection efficiency of miR-298 inhibitor in A549 cells. (B) Transient transfection efficiency of miR-298 mimic in A549 cells. Data are from three independent experiments. *P*-values were calculated using ANOVA with post hoc LSD test. Error bars represent SD. ** *P* < 0.01, *** *P* < 0.001 compared with the control group.

Supplementary Figure 4: Effects of miR-298 overexpression on c-Myc expression. (A) mRNA level of c-Myc in A549 cells treated with miR-298 mimics. (B) Protein level of c-Myc in A549 cells treated with miR-298 mimics. Data are from three independent experiments and were analysed using ANOVA with post hoc LSD test. Error bars represent SD. * *P* < 0.05, ** *P* < 0.01, *** *P* < 0.001 compared with the control group.
